# MiR-218通过抑制Robo1的表达影响肺癌细胞迁移侵袭

**DOI:** 10.3779/j.issn.1009-3419.2017.07.03

**Published:** 2017-07-20

**Authors:** 平 陈, 云龙 赵, 英杰 李

**Affiliations:** 100048 北京，解放军总医院第一附属医院 First Affiliated Hospital of PLA General Hospital, Beijing 100048, China

**Keywords:** 肺肿瘤, miR-218, Robo1, 迁移, 侵袭, Lung neoplasms, miR-218, Robo1, Migration, Invasion

## Abstract

**背景与目的:**

本研究旨在探讨肺癌中miR-218的表达，研究miR-218在肺癌细胞中的功能及其可能的分子机制。

**方法:**

应用实时荧光定量PCR（qRT-PCR）检测15例肺癌组织和15例癌旁组织中miR-218的表达。在肺癌细胞A549中转染miR-218的抑制物（Anti-miR-218），在肺癌细胞HCC4006中转染miR-218的模拟物后，用Transwell实验检测细胞的迁移侵袭能力的变化。用Targetscan和MiRanda软件预测miR-218的可能靶点，转染miR-218的抑制物及模拟物后用qRT-PCR和Western blot检测Robo1的mRNA和蛋白表达水平。用双荧光素酶报告基因方法鉴定miR-218和Robo1的调控关系。用Anti-miR-218、miR-218模拟物或阴性对照与Si-Robo1或Si-NC同时转染细胞，应用Transwell实验检测转染后细胞的侵袭迁移能力的变化。

**结果:**

与癌旁组织比较，肺癌组织中miR-218在肺癌组织中表达水平显著降低（*P* < 0.01）。在A549细胞中转染miR-218的抑制物，能够显著降低miR-218的表达，促进了细胞的迁移侵袭。在HCC4006中转染miR-218的模拟物能够显著提高miR-218的表达，同时抑制了细胞的迁移侵袭能力。利用生物信息学预测出在Robo1的3′UTR区有miR-218的结合位点，双荧光素酶报告基因实验进一步证实miR-218能够调控Robo1的转录活性。抑制miR-218能够提高Robo1的表达；过表达miR-218显著降低Robo1的表达，且miR-218能够通过调控Robo1影响细胞的迁移侵袭。

**结论:**

MiR-218在肺癌组织中呈现低表达状态，miR-218可能是通过抑制Robo1的表达抑制肺癌细胞侵袭迁移。

## Abstract (en)

**Background and objective:**

To explore the function and the potential molecular mechanism of miR-218 in lung cancer cell.

**Methods:**

The expression of miR-218 mRNA was determined by real-time PCR in lung cancer tissues, adjacent tissues and lung cancer cells. Transwell assay was used to detect the migration and invasion of A549 cell after transfected with Anti-miR-218 or negative control and HC4006 cell after transfected with miR-218 mimics and miR-218 negative control. Targetscan and MiRanda were used to calculate the potential targets of miR-218 and Luciferase reporter assay was performed to identify that the Robo1 was one target genes of miR-218. Transwell assay was used to detect whether miR-218 regulated the invasion of lung cancer cell transfected with anti-miR-218 or negative control via Robo1.

**Results:**

The expression of miR-218 in the lung cancer tissues was significantly lower than that in the adjacent tissues (*P* < 0.05). Inhibition of miR-218 improved the migration and invasion of A549 cell. Overexpression of miR-218 suppressed the migration and invasion of HCC4006 cell. The co-transfection of anti-miR-218 or miR-218 mimics and the Robo1 3′UTR increased or reduced the luciferase activity of Robo1 compared with the control group (*P* < 0.05). Inhibition of miR-218 and Robo1 recovered the invaded cells of A549. Overexpression of miR-218 and inhibition of Robo1 reduced the number of the invased cells of HCC4006. These results suggested that miR-218 banded Robo1 directly and inhibited lung cancer cell invasion by targeting Robo1.

**Conclusion:**

MiR-218 inhibited the migration and invasion of lung cancer cells through regulating Robo1 expression.

肺癌是死亡率最高的癌症之一，肺癌分为非小细胞肺癌和小细胞肺癌，其中80%属于非小细胞肺癌^[1]^。肺腺癌属于非小细胞肺癌，其侵袭和转移是一个多步骤、多基因参与的复杂过程，在这一过程中有大量的基因发生改变。尽管早期诊断技术有了很大的发展，放化疗技术不断的进步，但肺癌的5年生存率仍然很低^[2]^。MicroRNA（miRNA）是一类非编码小RNA，长度大约为22个核苷酸左右，越来越多的研究证明miRNA与肿瘤的发生和发展存在着密切的相关性^[3]^。近年研究发现miR-218能在多种肿瘤中表达，包括肺癌、膀胱癌、鼻咽癌等，且发挥着癌基因或者抑癌基因的作用^[4-6]^，但其作用机制尚未清楚。因此，本实验研究miR-218的功能，寻找它的靶点，探究其在肺癌中的作用机制，以期为肺癌的治疗提供新的靶点和理论依据。

## 1 材料与方法

### 1.1 一般资料

选择2015年6月-2016年6月解放军总医院第一附属医院手术切除的15例肺腺癌组织及其相应的癌旁组织标本，年龄为32岁-77岁，中位年龄为53岁。按肿瘤-淋巴结-转移（tumor-node-metastasis, TNM）分期，Ⅰ期4例，Ⅱ期5例，Ⅲ期6例。所有患者手术前均未接受放疗、化疗及内分泌治疗。所有患者均己签署知情同意书，手术切除部分组织用于浸泡福尔马林中用于组织切片鉴定，其余组织马上放入液氮中存储待用。

### 1.2 试剂

肺腺癌细胞系A549、NCI-H1299、Calu-3、NCI-H2228、NCI-H1975、HCC4006、NCI-H23、NCI-H1435均购于ATCC，DMEM培养液购自Gibic公司，miR-218的抑制物（Anti-miR-218）和对照组（Negative control）；Robo-siRNA及对照组干扰片段均由上海吉玛有限公司合成。转染试剂Lipfectmine 2000购于Invitrogen公司，兔源性的一抗Robo1、E-cadherin、N-cadherin、鼠源性的一抗GAPDH抗体购自Santa Cruz公司，二抗均购自北京中杉金桥公司；TRIzol购自Invitrogen公司，反转录及PCR试剂盒购自TAKARA公司。Transwell小室购自美国corning公司。miRNA RT Kit和MiRNA qPCR kit购自上海天根生化公司。

### 1.3 方法

#### 1.3.1 细胞培养

所用的肺腺癌细胞均用DMEM培养液（含10%胎牛血清，100 U/mL青霉素和0.1 mg/mL链霉素）培养，置于37 ℃、体积为5%的CO_2_培养箱中。

#### 1.3.2 转染

将Anti-miR-218和negative control，miR-218模拟物及miR-218阴性对照，Robo1-SiRNA和对照Si-Con片段分别与Lip2000混匀，在室温条件下混匀静置20 min后，慢慢滴入培养液中混匀，共培养细胞4 h后，换成新鲜的正常培养液继续培养24 h，再用于后续实验。Anti-miR-218序列为：5′-ACAUGGUUAGAUCAAGCACAA-3′，Negtive control序列为：5′-UUGUACUACACAAAAGUACUG-3′。Robo1 siRNA序列为：Sense: 5′-CCCAAGATTGTCGATCAAdtdt-3′，Antisense：5′-UUGAUCGACAAUCUUGGGdtdt-3′。对照序列si-NC序列为：Sense：5′-AGCGCGCTTTGTAGGATTC-Gdtdt-3′；Antisense：5′-GCAATCCTACAAAGCGCGCTdtdt-3′。miR-218模拟物（mi R-218 mimics）序列为：5′-UUGUGCUUGAUCUAAC-CAUGU-3′，miR-218阴性对照（miR-218 negative control, miR-218NC）序列为：5′-UCACAACCUCCUAGAAAGAGUAGA-3′。

#### 1.3.3 qRT-PCR检测miRNA和mRNA的表达

用TRIzol提取经转染的A549，HCC4006细胞总RNA，分别测定RNA浓度及纯度。采用上海天根生化公司的miRNA RT Kit将miRNA逆转录为cDNA，按照上海天根生化公司的MiRNA qPCR kit说明进行miRNA的PCR反应及定量分析。mRNA定量分析按照TAKARA公司反转录和定量PCR试剂盒说明书操作。Robo1定量PCR引物序列：Forward：5′-CCTGTGTCTACAGACAGCAACATGA-3′，Reverse：5′-GCACTGGAGGTGGTGGAAGA-3′；β-actin Forward：5′-AGCGAGCATCCCCCAAAGTT-3′；Reverse：5′-GGGCACGAAGGCTCATCATT-3′。

#### 1.3.4 蛋白质印迹检测蛋白的表达

细胞转染培养24 h后，弃培养基，用PBS清洗细胞，加入蛋白裂解液RIPA，12, 000 rpm离心15 min，吸取上清液待测。用BCA试剂盒定量蛋白浓度。在10%的SDS-PAGE凝胶中进行电泳（电压100 V），之后在300 mA的电流下冰浴转膜。转膜后室温下用5%BSA-TBST封闭PVDF膜1 h，在4 ℃条件下孵育一抗（1:1, 000）过夜，0.1%的TBST洗3次，每次5 min。在室温条件下与二抗共孵育1 h，0.1%的TBST洗3次，每次5 min。用化学发光法来检测蛋白表达，试验重复3次。

#### 1.3.5 Transell实验检测细胞迁移及侵袭

转染的细胞经0.25%胰酶消化，用无血清DMEM培养基制成单细胞悬液，用细胞计数板计数。稀释后，取400 µL（约含1×10^5^个细胞）加入到Transwell上室中（孔径为8 μm），在下室中加入500 µL含有15%FBS的DMEM培养液，细胞继续培养20 h。用棉签擦去上室的上表面细胞，PBS清洗2遍，无水甲醇固定20 min；室温下用0.4%的结晶紫染液孵育2 h。在显微镜下拍照，每张膜随机选取上、中、下各3个视野，进行统计分析，每组设3个重复。侵袭实验唯一的区别在于在Transwell上室上表面涂有一层基质胶，能够模仿细胞外基质。其他步骤与迁移实验相同。

#### 1.3.6 萤光素酶报告基因载体构建

运用生物学信息软件预测miR-218的靶基因，发现*Robo1*为候选靶基因之一。提取A549细胞基因组DNA，通过PCR扩增Robo1全长的3′UTR序列，扩展引物分别添加*Xho* Ⅰ和*Not* Ⅰ酶切位点。*Xho* Ⅰ和*Not* Ⅰ双酶切PCR扩增产物，随后将扩增片段连接到psiCHECK-2载体上。连接产物转化感受态DH5α大肠杆菌，酶切鉴定正确的阳性克隆送测序。将构建好的psiCHECK-2-ROBO1-3′UTR与Neg contrtol及Anti-miR-218（100 nmol/L）按Lipofectamine 2000转染试剂盒说明书操作进行共转染A549；将构建好的psiCHECK-2-ROBO1-3′UTR与miR-NC及miR-218 mimics（100 nmol/L）组严格按Lipofectamine 2000转染试剂盒说明书操作进行共转染HCC4006，最后进行双荧光素酶检测。

### 1.4 统计学方法

数据用SPSS 13.0软件分析，用均数±标准差（Mean±SD）表示计量资料，组间比较采用*t*检验，*P*＜0.05为差异有统计学意义。

## 2 结果

### 2.1 MiRNA-218在肺癌组织和肺癌细胞株中的表达

通过定量PCR检测了15例肺癌组织和15例癌旁组织内miR-218的表达，结果显示肺癌组织中miRNA-218的表达明显低于癌旁组织（*P*＜0.05，图 1A）。对肺腺癌细胞株A549、NCI-H1299、Calu-3、NCI-H2228、NCI-H1975、HCC4006、NCI-H23、NCI-H1435中miRNA-218的检测发现细胞株A549中miRNA-218的表达高于其他细胞中miRNA-218的表达，而HCC4006细胞中miRNA-218的表达最低（图 1B）。

**1:**
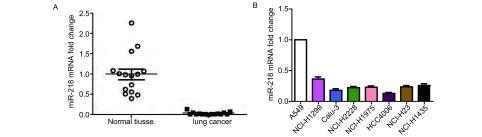
miRNA-218在组织和细胞中的表达情况。A：qPCR检测miR-218在肺癌组织及癌旁组织中的表达情况；B：qPCR检测miR-218在肺癌细胞系中的表达情况 Expression of miRNA-218 in tissues and cells. A: qPCR analysis of miR-218 expression in lung cancer tissues and adjacent tissues; B: qPCR analysis of miR-218 expression in lung cancer cell lines.

### 2.2 抑制miR-218对肺癌细胞株中的迁移侵袭的影响

在肺癌细胞系A549中转染Anti-miR-218的实验组及对照组（negative control），用定量PCR检测干扰后的效果，结果显示：细胞转染Anti-miR-218后，miR-218的表达明显降低，为对照组的0.27倍（*P*＜0.05，图 2A）。为了证实miR-218对于细胞迁移侵袭的影响，细胞转染后进行Transwell实验，结果显示转染Anti-miR-218组，细胞迁移数为（123.26±9.17），对照组胞迁移数为（76.62±6.81）（*P*＜0.05，图 2B-图 2C）；细胞侵袭数Anti-miR-218实验组为（102.83±11.67），对照组胞侵袭数为（62.43±8.44）（*P*＜0.05，图 2B-图 2C）。同时，我们在HCC4006细胞中转染miR-218的模拟物（miR-218 mimics），结果显示转染后，miR-218表达较对照组显著升高（*P*＜0.05，图 2D）；转染组细胞迁移数为（45.58±5.22），对照组胞迁移数为（73.31±8.95）（*P*＜0.05，图 2E-图 2F）；转染组细胞侵袭数为（26.84±3.67），对照组胞侵袭数为（56.53±5.46）（*P*＜0.05，图 2E-图 2F）。上述的迁移侵袭结果说明抑制miR-218能够显著增强A549细胞的迁移侵袭能力；过表达miR-218能够显著抑制HCC4006细胞的迁移侵袭能力。

**2:**
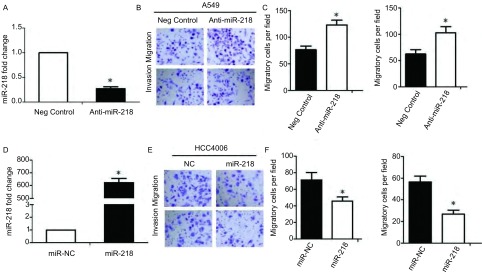
MiR-218影响肺癌细胞的迁移侵袭能力。A：qPCR检测在A549细胞中干扰miR-218后，miR-218的表达；B：干扰miR-218后，细胞的迁移侵袭实验（20×）；C：干扰miR-218后，A549细胞迁移侵袭数量的统计分析；D：qPCR检测在HCC4006细胞中转染miR-218 mimics后，miR-218的表达情况；E：转染miR-218 mimics后，HCC4006细胞的迁移侵袭实验（20×）；F：转染miR-218 mimics后，HCC4006细胞的迁移侵袭数量的统计分析。 MiR-218 affect the migration and invasion of lung cancer cells. A: qPCR analysis of miRNA-218 expression in A549 cell after transfected with Anti-miR-218; B: Transwell assay analysis of A549 cell after transfected with Anti-miR-218 (20×); C: Statistical analysis the migrated and invaded A549 cells after transfected with Anti-miR-218; D: qPCR analysis of miRNA-218 expression in HCC4006 cell after transfected with miR-218 mimics; E: Transwell assay analysis of HCC4006 cell after transfected with miR-218 mimics (20×); F: Statistical analysis the migrated and invaded HCC4006 cells after transfected with miR-218 mimics.

### 2.3 肺癌细胞中miR-218的靶基因预测

通过Targetscan和MiRanda软件预测miR-218的靶基因，我们发现miR-218能够识别*Robo1*基因的3′UTR区（图 3A）。我们应用qPCR和蛋白印迹方法，分别检测干扰miR-218后，A549肺癌细胞中Robo1的表达情况；过表达miR-218后，HCC4006细胞中Robo1的表达情况。结果显示Anti-miR-218组中Robo1的mRNA表达是对照组的2.87倍（图 3B，*P*＜0.05）；Western blot结果显示Anti-miR-218组中Robo1的蛋白表达量明显高于对照组（图 3C），结果提示我们抑制miR-218增加了A549细胞中Robo1的表达。在HCC4006细胞中转染miR-218 mimics，发现miR-218表达显著降低，为对照组的0.48倍（图 3D，*P*＜0.05）；Western blot结果显示转染组中Robo1的蛋白表达量明显低于对照组（图 3E）。我们进一步用双荧光素酶报告基因实验验证miR-218靶基因的准确性，将Anti-miR-218与psiCheck2-Robo1-3′UTR或对照质粒共转染A549细胞；将miR-218 mimics与psiCheck2-Robo1-3′UTR或对照质粒共转染HCC4006细胞培养，培养48 h后，检测荧光素表达水平。结果发现，在A549细胞中抑制miR-218能够显著增强Robo1的转录活性，而对照组的荧光素酶的表达不受影响（图 3F，*P*＜0.05）；在HCC4006细胞中过表达miR-218能够显著抑制Robo1的转录活性（图 3G，*P*＜0.05）。结果提示miR-218能与*Robo1*基因的3′UTR区有结合，影响Robo1的表达。

**3:**
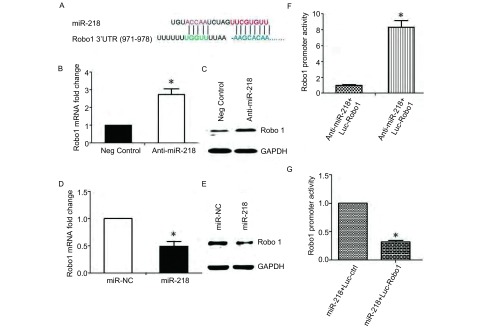
MiR-218靶基因的预测及验证。A：miR-218与Robo1的3’UTR结合示意图；B：干扰miR-218后，Robo1的mRNA变化情况；C：干扰miR-218后，Robo1蛋白的表达变化；D：qPCR检测在HCC4006细胞中转染miR-218 mimics后，Robo1的mRNA表达；E：在HCC4006细胞中转染miR-218 mimics后，Robo1的蛋白表达；F、G：双荧光素酶检测miR-218对Robo1转录活性的影响，其中F：干扰miR-218；G：过表达miR-218。 Predicting and verifying the target genes of miR-218. A: Schematic diagram for binding of miR-218 in 3'UTR of Robo1; B: qPCR analysis of Robo1 expression after Anti-miR-218 transfection; C: The protein level of Robo1 after Anti-miR-218 transfection; D: qPCR analysis of Robo1 expression in HCC4006 cell after transfected with miR-218 mimics; E: The protein level of Robo1 in HCC4006 cell after transfected with miR-218 mimics; F, G: Luciferase reporter assay analysis of the transcription activity of Robo1 in cells transfected with Anti-miR-218 (F) or over-expressed with miR-218 (G).

### 2.4 miR-218通过Robo1调控肺癌细胞的侵袭能力

为了验证miRNA-218是否通过Robo1来调控细胞的侵袭能力，我们在A549细胞中将侵袭实验分为4个组，分别为：Negative control+Robo1的Si-NC组为第1组，Anti-miRNA-218+Robo1的Si-NC组为第2组，Negative control+Si-Robo1组为第3组，Anti-miRNA-218+Si-Robo1组为第4组。每组分别共转染A549细胞，继续培养24 h后。再用Transwell小室按照细胞侵袭实验步骤进行操作。结果显示第2组与第1组比较，穿越Matrigel胶的细胞数明显增多，分别为[(102.82±11.67), (66.43±8.45)]（图 4A-图 4B，*P*＜0.05）；第3组细胞数为（44.36±7.02）与第1组比较明显减少（图 4A-图 4B，*P*＜0.05）；第4组细胞数为（58.62±9.15），与第1组比较没有统计学意义。在HCC4006中将侵袭实验也分为4组，分别为miR-NC+Si-NC组为A组，miRNA-218+Si-NC组为B组，miR-NC+Si-Robo1组为C组，miR-218+Si-Robo1组为D组。结果显示A组穿越Matrigel胶的细胞数（86.53±11.58），B组穿越Matrigel胶的细胞数（47.87±9.05），C组细胞数为（51.56±7.89），D组细胞数为（78.55±8.94）。B组及C组分别与A组比较差异显著，有统计学意义（图 4C-图 4D，*P*＜0.05）；D组与A组比较没有显著差异。提示miR-218是通过调控Robo1的表达来影响细胞的侵袭。

**4:**
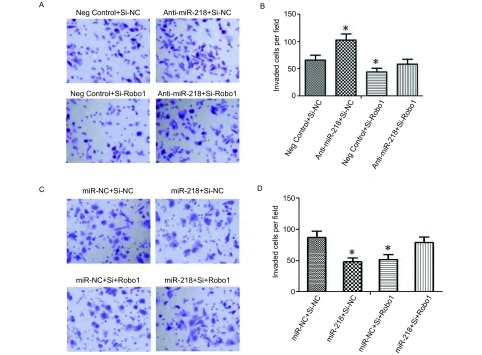
MiR-218通过调控Robo1影响肺癌细胞的侵袭能力。A：Anti-miR-218和Si-Robo1及其对照组后，A549细胞侵袭能力的改变（20×）；B：侵袭细胞的统计结果；C：转染miR-218 mimics和Si-Robo1及其对照组后，HCC4006细胞侵袭能力的改变（20×）；D：HCC4006细胞侵袭能力统计结果分析。 MiR-218 can influence the invasion ability of lung cancer cells by regulating Robo1 expression. A: Comparing the invasion ability of A549 cell transfect with Anti-miR-218 or Si-Robo1 to their control (20×); B: Statistical analysis of invaded cells in A549; C: Comparing the invasion ability of HCC4006 cell transfect with Anti-miR-218 or Si-Robo1 to their control (20×); D: Statistical analysis of invaded cells in HCC4006.

## 3 讨论

肺癌是世界上最常见的恶性肿瘤之一。随着现代化在我国进程的快速发展，肺癌的发病率呈持续升高的态势。尽管目前临床上对肿瘤诊治水平有了很大的进步，但针对肺癌的有效治疗效果不显著^[7]^。由于肺癌的发生发展机制目前并不清楚，患者生存预后较差。因此，深入研究肺癌发生发展的分子机制十分必要。

MiRNA参与到肿瘤的发生发展已被证实^[8]^。其中，miR-218不仅在多种组织中（如脂肪、皮肤等）发挥着重要的作用^[9-11]^。在人类多种恶性肿瘤中miR-218能够影响肿瘤细胞的增殖和凋亡，起着抑癌基因的作用^[4-6]^。本研究通过qPCR证明了miR-218在肺癌患者组织中是低表达的。已有的研究^[12, 13]^发现在人类多种肿瘤中miR-218均为低表达，并推测miR-218起着抑癌基因的作用。在肺癌中，有研究^[4]^认为miR-218低表达的是由于其甲基化作用的结果，并且miR-218是影响NSCLC生存和无复发预后的独立因素。本研究在miR-218高表达的A549细胞中抑制miR-218的表达发现细胞的转移侵袭功能增强，这与以前的研究^[14, 15]^结论相似。

MiR-218靶基因众多，包括Ecop、PAXILLIN、Robo1等，意味着miR-218有多种的调控通路。有研究已证实miRNA-218能通过调控Robo1参与多种癌症细胞侵袭和转移^[6, 16]^。在结直肠癌细胞中，miR-218可通过下调Bmi1的表达抑制细胞的增殖，促进细胞凋亡^[17]^。Wu等^[4]^研究发现miR-218通过靶向调控*Paxillin*基因表达来影响口腔鳞癌患者的生存及预后。miR-218可直接结合于ECOP的mRNA 3′-UTR，抑制ECOP基因表达，从而达到抑制胶质瘤细胞增殖的结果^[18]^。可见miR-218可参与多种调控肿瘤转移的机制中，但在肺癌中的调控机制尚不明确。

Robo1是轴突导向性受体，近来研究发现Robo1参与调控肿瘤的侵袭和转移^[12, 19, 20]^。我们用软件预测确实显示*Robo1*为miR-218靶基因之一，因此我们推测miR-218-Robo1通路可能也参与调控肺癌的侵袭和转移。我们通过外源性miR-218的抑制物瞬时干扰A549细胞中miR-218的表达，显著抑制了Robo1的表达。然后通过双荧光素酶报告基因实验进一步验证miR-218能够调控Robo1的转录活性，这些结果都明在肺癌细胞A549中Robo1是miR-218下游的靶基因之一。用miR-218抑制剂和Robo1的SiRNA同时转染细胞，发现抑制了miR-218增强细胞侵袭的能力，进一步证明了miR-218通过抑制Robo1的表达从而调控肿瘤细胞的迁移侵袭功能。在乳腺癌、胃癌和鼻咽癌等癌症中，同样存在miR-218-Robo1通路，且参与了调控肿瘤细胞的转移^[6, 17, 21]^。

综上所述，*Robo1*基因是miR-218的靶基因之一，miR-218能够通过调控*Robo1*基因的表达影响肺癌细胞的迁移侵袭，提示miR-218可通过调控下游靶基因来影响肺癌的发生发展。因此，miR-218可作为肺癌分子治疗中潜在的靶点，为临床治疗提供一个新思路及理论基础。
